# Monitoring the progress of health-related sustainable development goals (SDGs) in Brazilian states using the Global Burden of Disease indicators

**DOI:** 10.1186/s12963-020-00207-2

**Published:** 2020-09-30

**Authors:** Daiane Borges Machado, Júlia Moreira Pescarini, Dandara Ramos, Renato Teixeira, Rafael Lozano, Vinicius Oliveira de Moura Pereira, Cimar Azeredo, Rômulo Paes-Sousa, Deborah Carvalho Malta, Mauricio L. Barreto

**Affiliations:** 1 Center of Data and Knowledge Integration for Health (Cidacs), Oswaldo Cruz Foundation, Salvador, Brazil; 2 Centre for Global Mental Health, London School of Hygiene & Tropical Medicine (LSHTM), London, UK; 3 Institute of Collective Health (ISC), Federal University of Bahia (UFBA), Salvador, Brazil; 4 Public Health Graduate Program, School of Medicine, Federal University of Minas Gerais, Belo Horizonte, Brazil; 5 School of Medicine, Institute for Health Metrics and Evaluation, University of Washington, Seattle, Washington USA; 6 Brazilian Institute of Geography and Statistics (IBGE), Rio de Janeiro, Brazil; 7 René Rachou Institute, Fiocruz Minas, Belo Horizonte, Brazil; 8 Escola de Enfermagem, Departamento Materno Infantil e Saúde Pública, Universidade Federal de Minas Gerais, Belo Horizonte, Brazil

**Keywords:** Health disparities, Poverty, Inequalities, Middle-income countries, Health indicators

## Abstract

**Background:**

Measuring the Global Burden of Disease (GBD) has been the key to verifying the evolution of health indicators worldwide. We analyse subnational GBD data for Brazil in order to monitor the performance of the Brazilian states in the last 28 years on their progress towards meeting the health-related SDGs.

**Methods:**

As part of the GBD study, we assessed the 41 health-related indicators from the SDGs in Brazil at the subnational level for all the 26 Brazilian states and the Federal District from 1990 to 2017. The GBD group has rescaled all worldwide indicators from 0 to 100, assuming that for each one of them, the worst value among all countries and overtime is 0, and the best is 100. They also estimate the overall health-related SDG index as a function of all previously estimated health indicators and the SDI index (Socio-Demographic Index) as a function of per capita income, average schooling in the population aged 15 years or over, and total fertility rate under the age of 25 (TFU25).

**Results:**

From 1990 to 2017, most subnational health-related SDGs, the SDG and SDI indexes improved considerable in most Brazilian states. The observed differences in SDG indicators within Brazilian states, including HIV incidence and health worker density, increased over time. In 2017, health-related indicators that achieved good results globally included the prevalence of child wasting, NTD, household air pollution, conflict mortality, skilled birth attendance, use of modern contraceptive methods, vaccine coverage, and health worker density, but poor results were observed for child overweight and homicide rates. The high rates of overweight, alcohol consumption, and smoking prevalence found in the historically richest regions (i.e., the South and Southeast), contrast with the high rates of tuberculosis, maternal, neonatal, and under-5 mortality and WASH-related mortality found in the poorer regions (i.e., the North and Northeast).

**Conclusions:**

The majority of Brazil’s health-related SDG indicators have substantially improved over the past 28 years. However, inequalities in health among the Brazilian states and regions remain noticeable negatively affecting the Brazilian population, which can contribute to Brazil not achieving the SDG 2030 targets.

## Background

Monitoring inequalities has emerged as a priority for health post-2015. As the Millennium Development Goals (MDGs) have focused mainly on fighting poverty, promoting development, and reducing inequalities between countries, the post-2015 sustainable development goals (SDG) stress “leaving no one behind”—with goal 10 specifically calling for the reduction of inequality not only between but within countries [1, 2].

Across its 17 goals, 169 targets and 241 indicators, the SDG agenda covers issues such as poverty eradication, food security and agriculture, health, education, gender equality, inequality reduction, energy, water and sanitation, sustainable production and consumption patterns, climate change, sustainable cities, protection and sustainable use of oceans and terrestrial ecosystems, inclusive economic growth, infrastructure and industrialization, governance, and means of implementation [2, 3]. Health is highlighted by goal 3, which aims to “Ensure healthy lives and promote well-being for all at all ages.” However, health-related targets are also present across other goals within the agenda, contemplating various topics such as nutrition, sanitation, and water and air pollution.

Across three dimensions and several indicators, the WHO guidelines propose that in low-and-middle-income-countries, the within-country monitoring should focus on those aspects that are particularly relevant to each of those countries, and that by drawing upon the conclusions of monitoring inequalities in health, policies could then be tailored to ensure that SDG progress is happening in an equity promoting manner [4].

Brazil provides an interesting scenario for this type of within-country evaluation. It is a middle-income country that over the past 50 years has gone through significant economic and structural changes. Despite being classified as the 9th top economy in the world and the 2nd biggest in the western hemisphere in terms of nominal Gross domestic product (GDP), Brazil has one of the highest levels of income inequality [5, 6] and also large regional inequalities [7]. This inequality has deep historical and regional roots and remains a current issue, leading to unequal access to goods across the Brazilian population.

The country has an extension of 8.5 million km^2^ and a population of 209.3 million people living in 26 states and the Federal District. These states are grouped into five geographic macro-regions: North, Northeast, Central-west, Southeast, and South. States in the regions of the South and Southeast are where the largest cities São Paulo and Rio de Janeiro can be found. They are highly industrialised, with better infrastructure and healthcare, compared with those in the North and Northeast. The demographic and epidemiological changes in Brazil have not occured uniformly across states, resulting in different patterns of the population dynamics and regional disparities in health and corresponding burdens on health systems [8–10].

The Brazilian Network of GBD researchers (GBD Brazil Project) have shown that although the health situation in Brazil improved greatly from 1990 to 2016, the advances and the burden of disease varied significantly across states [9]. Therefore, to achieve health equity and to guide resource allocation towards local health problems, it is important to analyse sub-national data on health and its determinants [11–14]. In order to operationalize such an endeavour, the GBD consortium has defined 41 integrated and intersectoral health indicators related to health, including their social and environmental determinants [15].

Estimating subnational health-related SDGs progress in Brazil through the GBD indicators can identify high-performing and low-performing states and therefore inform whether policies across Brazilian states are being directed toward those in greatest need.

Previous studies using subnational data in many countries have helped guide governments in resource allocation towards national health problems [11–14]; however, studies focusing on within-country analysis and monitoring of health-related SDGs progress are largely from Europe and North America. To the best of our knowledge, there are no investigations measuring the performance of the SDG indicators related to health in the Brazilian states. Therefore, we analyse subnational Global Burden of Disease GBD data for Brazil in order to monitor the performance of the Brazilian states over the last 28 years on their progress towards meeting the health-related SDGs. Additionally, we report on the trends in inequalities in health among the states and discuss the health policy implications of our findings.

## Methods

We analysed subnational GBD indicators for Brazil at the state level from 1990 to 2017. Our network of collaborators—GBD Brazilian Network of researchers (GBD Brazil Project)—adapted the GBD methodology for the Brazilian context [9]. Selected indicators included disaster mortality, child stunting, child wasting, child overweight, maternal mortality ratio, skilled birth attendance, under-5 mortality, neonatal mortality, HIV incidence, tuberculosis (TB) incidence, malaria incidence, hepatitis B incidence, prevalence of neglected tropical diseases (NTDs), premature mortality due to non-communicable diseases deaths (NCD), suicide mortality rate, alcohol use, road injury mortality, proportion of woman using contraceptives, adolescent birth rate, universal health coverage (UHC) index, mortality attributable to air pollution, mortality attributable to WaSH (water, sanitation, and hygiene), poisoning mortality, smoking prevalence, vaccine coverage, health worker density, intimate partner violence, sexual violence (non-intimate partner), water, sanitation, hygiene, household air pollution, disease burden attributable to occupational risks, mean PM2.5, homicide, conflict and terrorism mortality, physical violence prevalence, sexual violence prevalence, child sexual abuse, and certificate death registration.

### Data sources and indicators

This study used estimates of all-cause mortality, cause-specific mortality and morbidity, combined health loss, risk factor exposure, and attributable burden of disease by age and sex. The Global Burden of Disease, from the *Institute of Health Metrics and Evaluation* (IHME), produced and made available national and subnational data, from 1990 to 2017, for 195 countries and territories. Subnational estimates from GBD 2017 cover countries with populations of over 200 million (i.e., China, India, United States, Indonesia, and Brazil) [16–18], as well as additional countries which have requested and undertaken subnational analyses collaboratively with the GBD study such as Japan, Ethiopia, Iran, Kenya, Mexico, Norway, Russia, South Africa, and Sweden [9, 16]. The data sources used by the GBD study to generate Brazilian estimates contain more than 2000 records, including administrative data (e.g., birth, deaths, and national diseases surveillance registries), primary collected data, data from epidemiological studies, and scientific literature reviews. The complete list of datasets and the results are available on the Global Health Data Exchange website (http://ghdx.healthdata.org/).

In this study, we analysed the Brazilian national and subnational data (at the state level) estimated by GBD from 1990 to 2017. We analysed 40 health-related SDG indicators for the 26 Brazilian states and the Federal district currently monitored by the GBD group and Brazilian GDB collaborators [9]. Specific indicators include those for health outcomes, health services, environmental, occupational, and behavioural risks with well-established causal connections to health. These are listed in Table 1, including name, description, and Brazilian target. Methods for estimating each indicator are included in other publications [16, 17, 19].

**Table 1:** Health-related SDG indicators according to GBD, description, and 2030 targets for Brazil

Goals and health related SDG indicator	Description	Brazilian target
**Goal 1: End poverty in all its forms everywhere**		
1.5.1: Disaster mortality	Age-standardised death rate due to exposure to forces of nature (per 100,000 population)	Undefined
**Goal 2: End hunger, achieve food security and improved nutrition, and promote sustainable agriculture**		
2.2.1: Child stunting	Prevalence (%) of stunting (height for age < -2 standard deviation from the median of the World Health Organization (WHO) Child Growth Standards) among children under 5 years of age.	Eliminate
2.2.2a: Child wasting	Prevalence of wasting (weight for height < -2 standard deviation from the median of the WHO Child Growth Standards) among children under 5 years of age.	Eliminate
2.2.2b: Child overweight	Prevalence of overweight (weight for height > +2 standard deviation from the median of the WHO Child Growth Standards) among children aged 2 to 4 years of age.	Eliminate
**Goal 3: Ensure healthy lives and promote well-being for all at all ages**		
3.1.1: Maternal mortality ratio	Maternal mortality ratio (MMR) defined as the number of maternal deaths among woman aged 15–49 years old during a given time period per 100,000 live births during the same period.	Reduce to 30 deaths by 100,000 live births (70 deaths per 100,000 live births)
3.1.2: Skilled birth attendance	Percentage of births attended by skilled health personnel (doctors, nurses, or midwives).	No specific target
3.2.1: Under-5 mortality	Probability of a child born dying before reaching the age of 5 years, in a specific year, expressed per 1000 live births.	Reduce to 8 deaths by 1000 live births (< 25 per 1000 live births)
3.2.2: Neonatal mortality	Probability of a child born dying in the first 28 days of life, in a specific year, expressed per 1000 live births.	Reduce to 5 deaths by 1000 live births (< 12 per 1000 live births)
3.3.1: HIV incidence	Age-standardised rate of new HIV infections per 1000 individuals.	Eliminate the epidemics of the AIDS
3.3.2: TB incidence	Age-standardised number of new tuberculosis (TB) cases per 100,000 population each year.	Eliminate the epidemics of the disease
3.3.3: Malaria incidence	Age-standardized rate of malaria per 1000 population each year.	Eliminate the epidemics of the disease
3.3.4: Hepatitis B incidence	Age-standardized rate of new cases of hepatitis B per 100,000 people at risk each year.	Eliminate the epidemics of the disease
3.3.5: Prevalence of NTDs	Age-standardised prevalence of 15 neglected tropical diseases (NTDs) in %: Human African Trypanosomiasis, Chagas disease, cystic echinococcosis, cysticercosis, dengue, food-borne trematodiases, Guinea worm, intestinal nematode infections, leishmaniasis, leprosy, lymphatic filariasis, onchocerciasis, rabies, schistosomiasis, and trachoma.	No specific target
3.4.1: Premature mortality due to NCD	Age-standardized death rate from cardiovascular diseases, cancers, diabetes and chronic respiratory disease, between the ages of 30 to 70 years per 100,000 population.	Reduce NCD in one third based on 2015 data.
3.4.2: Suicide mortality rate	Age-standardized suicide rate per 100,000 population each year.	No specific target
3.5.2: Alcohol use	Risk-weighted prevalence of alcohol consumption, as measured by the SEV for alcohol use, %	No specific target
3.6.1: Road injury mortality	Age-standardised death rate due to road injuries per 100,000 population.	Reduce deaths and injuries by half based on 2015 data.
3.7.1: Proportion of woman using contraceptives	Proportion of women of reproductive age (15–49 years) who have their need for family planning satisfied with modern methods, %	Assure universal availability of contraceptives, planning, education, and information on reproductive health.
3.7.2: Adolescent birth rate	Number of live births per 1000 women aged 10–14 years and women aged 15–19 years.	Assure universal availability of contraceptives, planning, education, and information on reproductive health.
3.8.1: Universal health coverage (UHC) index	Coverage of essential health services, as defined by a universal health coverage index of the coverage of nine tracer interventions and risk-standardised death rates from 32 causes amenable to personal health care	No specific target – Reduce waiting time for surgeries, access to medicine and catastrophic cost with medicine.
3.9.1: Mortality attributable to air pollution	Age-standardised death rate attributable to household air pollution and ambient air pollution, per 100 000 population	Reducing by half the proportion of untreated effluent discharge
3.9.2: Mortality attributable to WaSH	Age-standardised death rate attributable to unsafe WaSH, per 100,000 population	No specific target
3.9.3: Poisoning mortality	Age-standardised death rate due to unintentional poisonings, per 100,000 population	No specific target
3.a.1: Smoking prevalence	Age-standardised prevalence of daily smoking in populations aged 10 years and older, %	No specific target
3.b.1: Vaccine coverage	Coverage of eight vaccines, conditional on inclusion in national vaccine schedules, in target populations, %	100% coverage
3.c.1: Health worker density	The number of physicians, nurses or midwives, and pharmacists per 1,000 population in a given area.	No specific target
**Goal 5: Achieve gender equality and empower all women and girls**		
5.2.1: Intimate partner violence	Age-standardised prevalence of women aged 15 years and older who experienced physical or sexual violence by an intimate partner in the past 12 months, %	No specific target
5.2.2: Sexual violence (non-intimate partner)	Age-standardised prevalence of women aged 15 years and older who experienced physical or sexual violence by persons other than an intimate partner, in the previous 12 months.	No specific target
**Goal 6: Ensure availability and sustainable management of water and sanitation for all**		
6.1.1: Water	Risk-weighted prevalence of populations using unsafe or unimproved water sources, as measured by the SEV for unsafe water, %	Achieve universal coverage and equitable access to clean water (100%).
6.2.1a: Sanitation	Risk-weighted prevalence of populations using unsafe or unimproved sanitation, as measured by the SEV for unsafe sanitation, %	Achieve universal coverage of adequate and equitable sanitary facilities (100%).
6.2.1b: Hygiene	Risk-weighted prevalence of populations without access to a handwashing facility, as measured by the SEV for unsafe hygiene, %	Achieve universal coverage of adequate and equitable sanitary facilities (100%).
**Goal 7: Ensure access to affordable, reliable, sustainable, and modern energy for all**		
7.1.2: Household air pollution	Risk-weighted prevalence of household air pollution, as measured by the SEV for household air pollution, %	Achieve universal and affordable access to clean and modern sources of energy (100%).
**Goal 8: Promote sustained, inclusive, and sustainable economic growth, full and productive employment, and decent work for all**		
8.8.1: Disease burden attributable to occupational risks	Age-standardised all-cause DALY rate attributable to occupational risks per 100,000 population	No specific target—reduce vulnerability situation of workers, including informality, legislation, and working conditions.
**Goal 11: Make cities and human settlements inclusive, safe, resilient, and sustainable**		
11.6.2: Mean PM2.5	Population-weighted mean levels of PM2·5, μg/m^3^	No specific target—reduce the negative impact of pollution in cities.
**Goal 16: Promote peaceful and inclusive societies for sustainable development, provide access to justice for all, and build effective, accountable, and inclusive institutions at all levels**		
16.1.1: Homicide	Age-standardised death rate due to interpersonal violence per 100,000 population	Reduce all forms of violence and reduce in one third the rates of femicide and children, adolescents, young, blacks, indigenous and LGBT population base on 2015 rates.
16.1.2: Conflict and terrorism mortality	Death rate due to conflict and terrorism per 100 000 population	Reduce all forms of violence and reduce in one third the rates of femicide and children, adolescents, young, blacks, indigenous and LGBT population base on 2015 rates.
16.1.3a: Physical violence prevalence	Number of persons who have been victim of physical violence in the previous 12 months, as a share of the total population. NÃO ENCONTREI	Reduce all forms of violence and reduce in one third the rates of femicide and children, adolescents, young, blacks, indigenous and LGBT population base on 2015 rates.
16.1.3c: Sexual violence prevalence	Age-standardised prevalence of physical or sexual violence experienced by populations in the past 12 months, %	Reduce all forms of violence and reduce in one third the rates of femicide and children, adolescents, young, blacks, indigenous and LGBT populations base on 2015 rates.
16.2.3: Child sexual abuse	Age-standardised prevalence of women and men aged 18–29 years who experienced sexual violence by age 18 years, %	No specific target
**Goal 17: Strengthen the means of implementation and revitalise the global partnership for sustainable development**		
17.19.2c: Cert Death Reg	Well-certified deaths by a vital registration system among a country’s total population, %	No specific target

*DALY* disability-adjusted life-year, *GBD* Global Burden of Disease, *NCDs* non-communicable diseases, *SDG* sustainable development goal, *SEV* summary exposure value, *WaSH* water, sanitation, and hygiene, *PM2·5* fine particulate matter smaller than 2.5 μm. Detailed descriptions of the data and methods for estimating each indicator are included in other publications [18–20].

In order to compare the overall health results for the Brazilian states over time, we assessed the overall health-related SDG index as a function of the 40 health-related indicators for each state by year and, to evaluate the development status of the Brazilian states correlated with health outcomes, we assessed the sociodemographic index (SDI index) as a function of per capita income, the average schooling in the population aged 15 years or older, and the total fertility rate under the age of 25 (TFU25) [21]. Similar to the method used to calculate the Human Development Index (HDI), the SDG index and SDI index were both calculated as the geometric mean of all the indicators included and were analysed separately. In order to do this, the GBD first rescaled each indicator component from 0 to 100, where the 0 was the worst value and 100 the best value that was given in all 195 countries covered by GBD, over the entire period, from 1990 to 2017. Then, for each index (i.e., SDG index and SDI index), they calculated the mean value of all indicator component values for a given year for Brazil and each Brazilian state. Further details on the SDG and SDI methodology used for Brazil can be found in previous publications [9, 20].

In order to compare the overall health results for the Brazilian states over time, we included SDG Index and SDI to account for more specific sociodemographic characteristics. To explore further the progress of state-level indicators towards health equality in Brazil, the yearly coefficient of variability (standard deviation/mean) for each indicator was estimated, from 1990 to 2017. We used boxplots per year to describe the trends of the indicators with the largest standard deviation and the largest variability coefficient within states in the same period. Finally, we related the GBD estimates with the targets for 2030 adjusted for Brazil [22].

### Sensitivity analysis

As some Brazilian registry systems are often completed up to 2 years after the actual events occur (e.g., mortality data and some diseases from the Brazilian notification system), we also produced a scaled 2016 heatmap figure and compared it with the preliminary results from 2017.

## Results

**Fig. 1:**
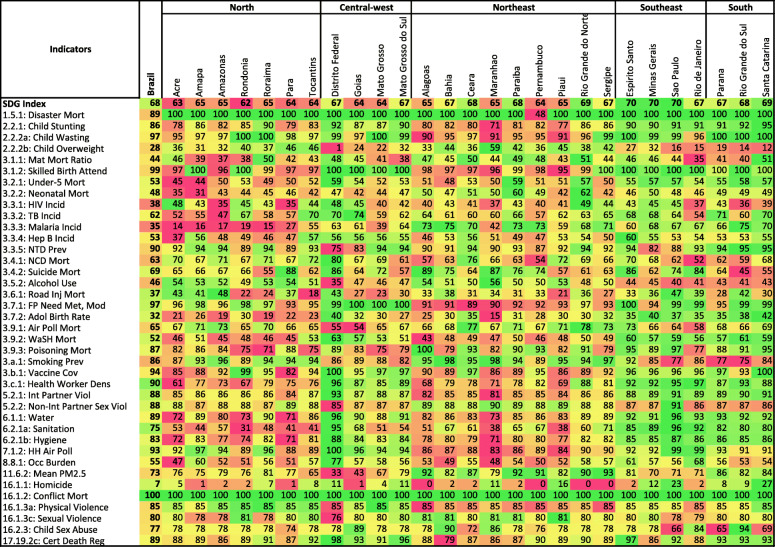
Performance on the health-related SDG index and individual health-related indicators in Brazilian States in 2017. The numbers represent the rescaled indicators and combined indexes on a scale of 0 to 100, with 0 representing the worst value for each indicator or index among all 195 countries covered by GBD from 1990 to 2017, and 100 representing the best value among them within the same period. The colours represent a scale from the best (dark green) to the worse (red) indicator or index values (rows) within all Brazilian States in 2017

After rescaling each of the 40 indicators from 0 to 100 globally, from 1990 to 2017, the five Brazilian regions showed strong disparities and different health patterns (Fig. 1).

**Fig. 2:**
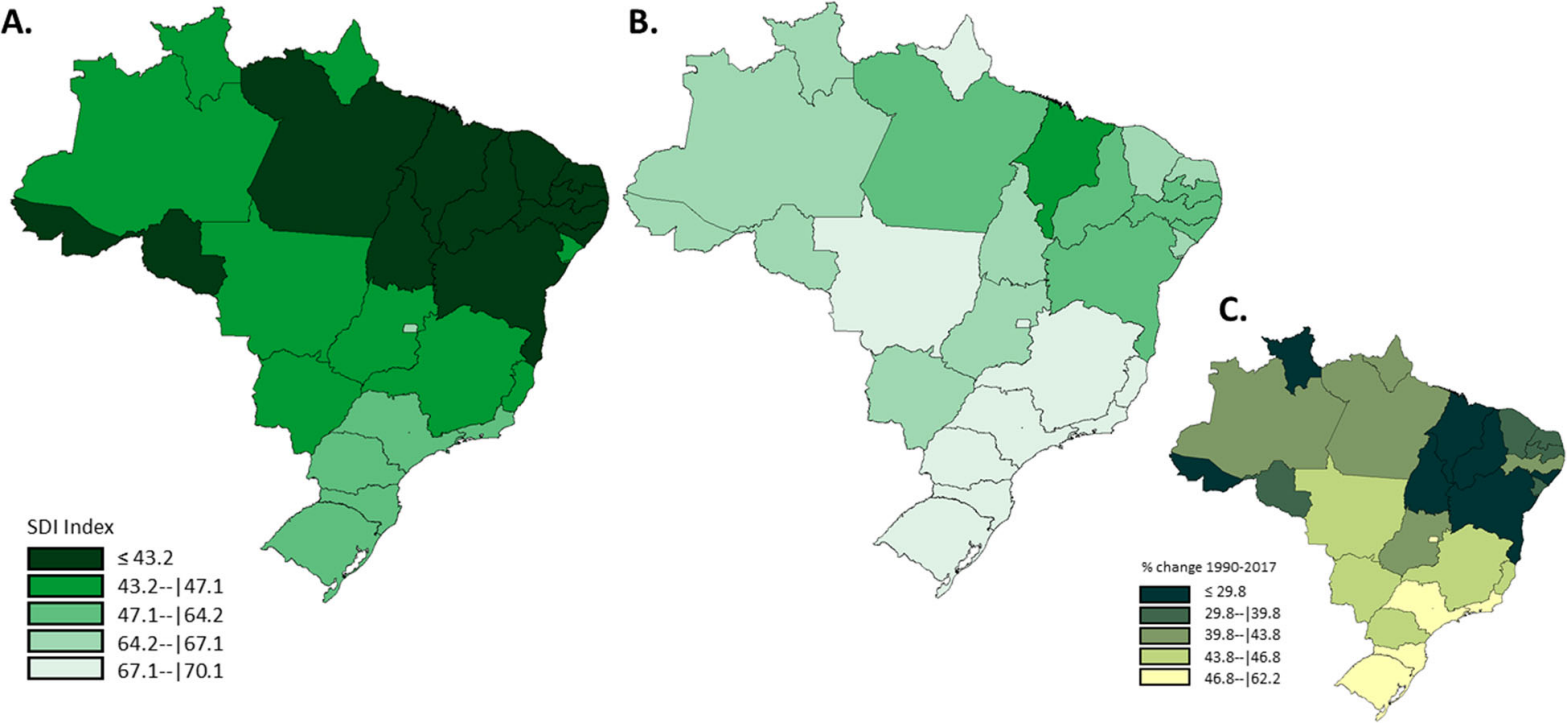
SDG index in 1990 (**a**), in 2017 (**b**), and percentage change from 1990–2017 (**c**). For fig. 2**a**, **b**, the color was coded by the quintile of SDG Index in 1990, were the dark green represents the worst and the light green the best SDG index. The same quintile distribution was used for SDG index distribution in 2017 for comparability purposes. The third map shows the percentage of change in SDG index from 1990 to 2017. The colors also represent the quintile distribution of percent change of SDG index, were the dark green represent the lowest changes and the yellow represent the largest changes during the period

**Fig. 3:**
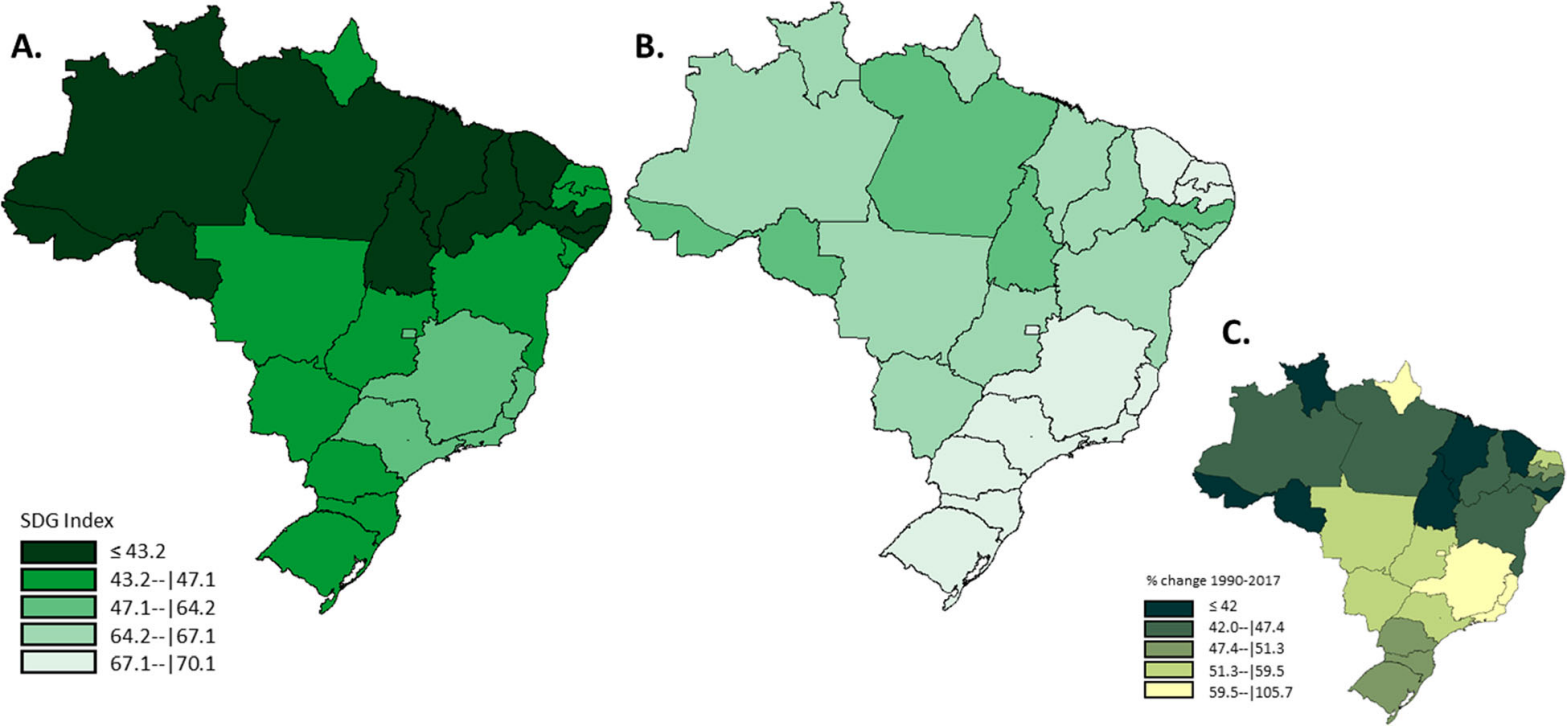
SDI index in 1990(**a**), in 2017(**b**), and percentage change in SDI index from 1990–2017(**c**). For fig. 3**a**, **b**, the color was coded by the quintile of SDI Index in 1990, were the dark green represents the worst and the light green the best SDI index. The same quintiles distribution was used for SDI index distribution in 2017 for comparability purposes. The third map shows the percentage of change in SDI index from 1990 to 2017. The colors also represent the quintile distribution of percent change of SDI index, were the dark green represent the lowest changes and the yellow represent the largest changes during the period

The combined SDG index (i.e., the geometric mean of the 40 health-related indicators) improved considerably from 1990 to 2017, overall and in all Brazilian States. The SDG index in Brazil varied from 45.7 (min-max values within states 44.6–46.7) to 67.6 (65.7–68.9) (Fig. 1). In 2017, apart from the majority of South and Southeast states of Brazil, only three states reached the second quintile of the SDG Index distribution (Fig. 2a, b). In addition, five states improved the SDG indexes substantially from 1990 to 2017 (up to a 105.7% increase) (Fig. 2c). Finally, the SDI index (i.e., the combined measure of per capita income, education, and fertility rate) also improved in all Brazilian states (Fig. 3a, b) in 2017; however, the greater relative increase was observed in the states located in the South and Southeast regions (Fig. 3c), the two richer areas of Brazil.

### Progress on health indicators

In 2017, Brazil showed overall good indicators for maternal and child health and access to healthcare. However, it also showed high rates of child overweight and homicides rates in comparison with other 195 countries analysed (Fig. 1). We observed a clear pattern of high child wasting in poor regions and high prevalence of child overweight in wealthier Brazilian regions (Fig. 1). While the prevalence of child stunting (chronic malnutrition) reached 18% in Maranhão (see Fig. 1 and Figure S1), the prevalence of overweight among children from 2–4 years old, which is largely concentrated in the South, Southeast, reached 49% in the Federal District (Brazilian capital). Eighty-eight percent (min-max 82–94%) of woman made use of contraceptive methods, and 100% (min-max 96–100%) of births were accompanied by skilled health professionals (Figure S1).

Although the overall performance of WaSH-related mortality in Brazil remains far from ideal, the mortality rate is substantially higher in the North and Northeast region of Brazil, which also accounts for the highest proportion of the population without access to clean water, sanitation, and hand washing facilities (see Fig. 1).

The GDB results indicate that not only TB but also HIV, malaria, and Hepatitis B are mostly found in the Northern states (Figure S2). The incidence of tuberculosis peaked in 1995/1996, gradually decreasing after that and stabilizing after 2010, with an incidence rate of 35.7/100,000 (min-max 18.6–80.0) in 2017 (data not shown). In the same year, the incidence of malaria was still substantially higher in the Amazon region compared to the other regions (see Fig. 3 and Figure S1).

In 2017, homicide rates in Brazil were high throughout the country; however, the North and Northeast states had the highest global homicide rates over the entire period. The highest homicide rates were in the state of Alagoas where they were found to be 4.4 times higher than the lowest in Rio Grande do Sul (53/100,000 vs. 12/100,000) (Figure S1). The high homicide rates in Brazil contrast with the absence of deaths due to conflict, which was null in 2017 (see Fig. 1, Figure S1).

Although there is an overall low NTD prevalence in Brazil compared to global results, the highest sum of NTDs prevalence was found in the Federal District (i.e., 0.25% of the population was affected by one of the 15 selected NTDs) (see Fig. 1, Figure S1). Brazilian indicators of healthcare also performed relatively well, including high vaccine coverage, the lowest coverage in 2017 was observed in the state of Para (82%), and there is a high health worker density, but which is also considerably poorer in North and Northeast states.

### Progress on reducing inequalities in health

**Fig. 4:**
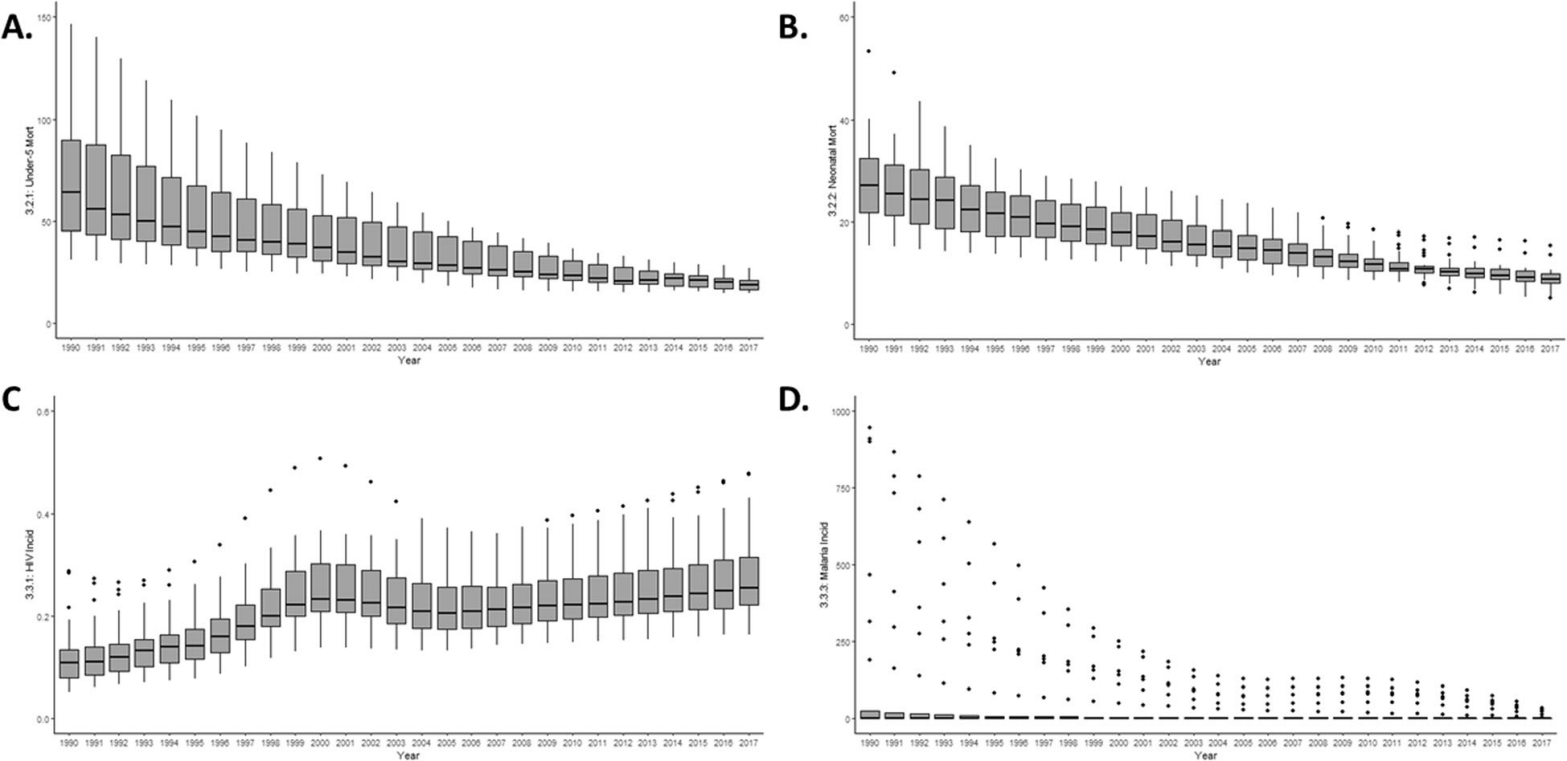
Trends of **a** under-5 mortality, **b** neonatal mortality, **c** HIV incidence, and **d** malaria incidence in 1990–2017. The boxplots represent the distribution of the four indicators across the Brazilian municipalities, for each year, from 1990–2017

Under-5 mortality rates decreased from 69.5/1000 live births in 1990 (min-max within Brazilian states 31.2–146.5) to 18.4 (min-max 14.4–23.7) in 2017 (Fig. 4a); neonatal mortality decreased from 26.3/1000 live births (min-max 15.4–61.4) to 8.4/1000 (min-max 5.1–13.6) in the same period (Fig. 4b). Inversely, the age-standardized rate of new HIV infections was more than doubled, from 0.16/1000 (min-max 0.05–0.29) in 1990 to 0.36/1000 (min-max 0.16–0.47) in 2017, and the distribution became heterogeneous among the Brazilian states (see Fig. 4c).

**Fig. 5:**
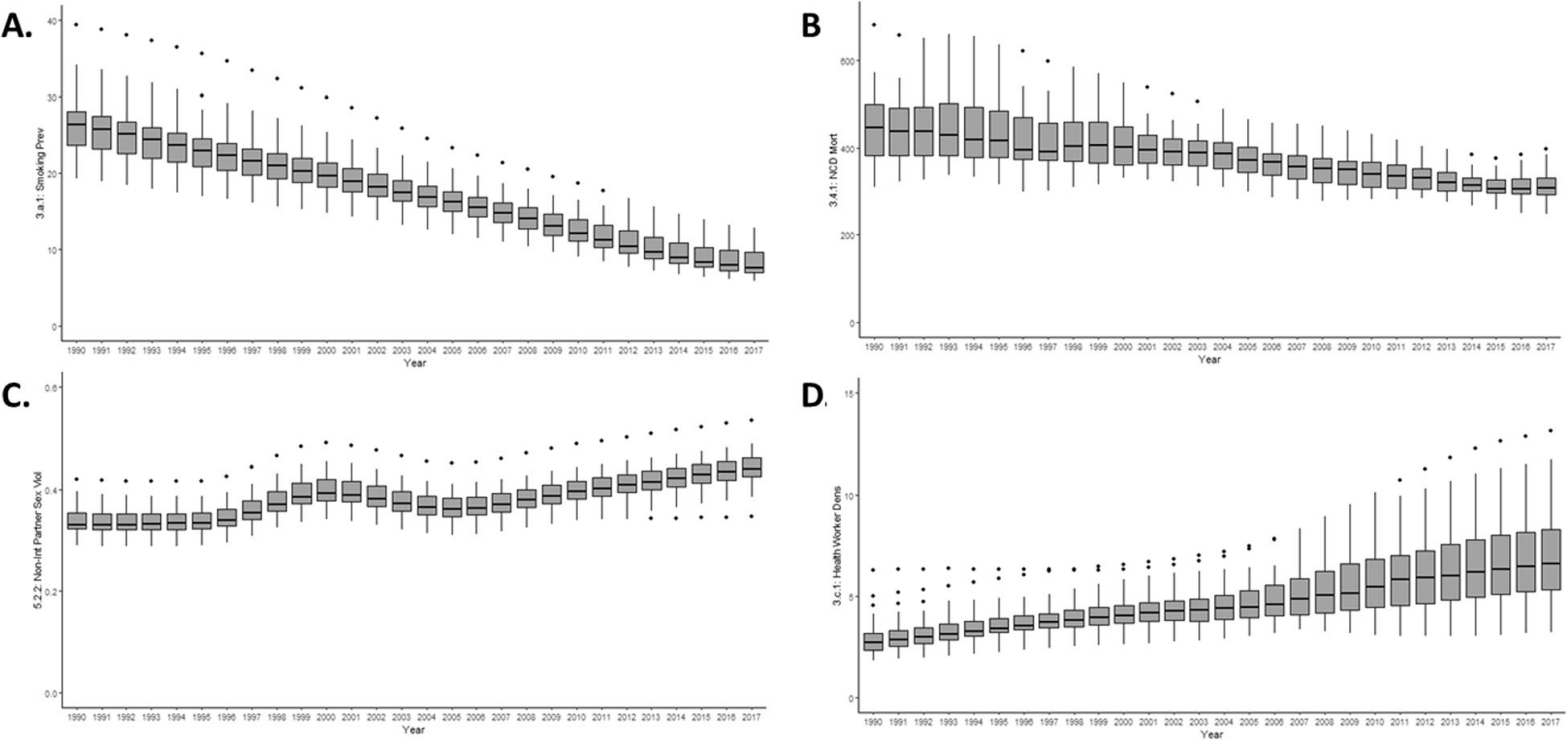
Trends of **a** smoking prevalence, **b** non-communicable diseases (NCD) mortality, **c** non-intimate partner sex violence, and **d** health workers density in 1990–2017. The boxplots represent the distribution of the four indicators across the Brazilian municipalities, for each year, from 1990–2017

The indicators with the largest heterogeneity in the distribution of indicators within Brazilian states (either in 2017 or as a mean of the entire 1990–2017 period) are under-5 and neonatal mortality, HIV and malaria incidence, smoking prevalence, NCD mortality, non-intimate partner sex violence, and health worker density (see Figs. 4 and 5). On the other hand, the indicators that varied less within states (in 2017 or in the entire period) are the population-weighted mean levels of PM2.5Mean, NCD mortality, and the percentage of well-certified death registrations.

The prevalence of smoking and NCD prevalence was also declined in all Brazilian states with substantial reductions in variability within states, with 8.8% (min-max 5.8–12.8) of the 2017 Brazilian population smoking and with an NCD mortality rate of 330/100,000 (min-max 290–398) (Fig. 5a, b). Non-intimate partner sex-related violence against women aged 15 or older increased during the period analysed (1990-2017) and in 2017 differences within regions remained large (0.43%, min-max 0.34–0.53) (Fig. 5c). Health worker density increased considerably during the period (from 3.4 in 1990 to 8.1/1000 in 2017) but inequalities within also increased, and the mean number of health professionals per person in 2017 was four times higher in Rio de Janeiro than in Acre (12/1000 vs. 3/1000) for example (Fig. 5d).

### Sensitivity analysis

Comparing the analysis of 2016 data (Figure S2) with the main analysis in 2017 (Fig. 1), we observed a relatively similar pattern of scaled indicators throughout the country (see Figure S2).

## Discussion

Overall, Brazilian states showed a considerable improvement in the Sustainable Development Goal (SDGs) indexes and indicators, from 1990 to 2017. However, substantial disparities in health-related indicators remain among the Brazilian states seem to have increased in the same period. The two richest regions of Brazil, the South and Southeast, not only had the highest SDG and SDI index in 1990 as also showed the greatest improvement from 1990 to 2017. Increased variability in indicators among states, such as HIV incidence and health worker density, suggests persistent inequalities on health among Brazilian states.

In 2017, Brazil obtained good results globally (≥ 90 in the rescaled indicators) for several indicators, including the prevalence of child wasting, NTD prevalence, household air pollution, conflict mortality rates, high proportion of skilled birth attendance, use of modern contraceptive methods, vaccine coverage, and health worker density. On the other hand, in the same year, almost all Brazilian states had very poor indicators for child overweight and strikingly high homicide rates. While the South and the Southeast regions of Brazil presented higher rates of overweight, alcohol consumption, and smoking prevalence, the poorer regions, the North and Northern, presented the worst rates of TB, maternal, neonatal, and under-5 mortality and WASH-related mortality.

The tenth SDG is to reduce inequalities among and within countries. This includes adopting social protection policies that progressively promote greater equality in health and in access to healthcare. One of the key components of the SDGs related to health is universal health coverage (UHC). Promoting equality in access to health is a core principle of UHC and is explicitly present in the SDGs 3.8 (“achieve universal health coverage, including financial risk protection, access to quality essential health-care services and access to safe, effective, quality, and affordable essential medicines and vaccines for all”) and 3.8.1 (coverage of essential health services, capturing the role of health systems in delivering effective interventions to improve a wide range of health outcomes). Although Brazil has been successful in implementing UHC since the 1990s, critical elements of inequalities in health within regions persist.

### Child and maternal health outcomes

Our results showed that child health outcomes in Brazil improved significantly between 1990 and 2017 while the disparities across state-level indicators fell also considerably. This report is consistent with those of previous studies on the progress of child health outcomes and childhood mortality [23–25]. As pointed out in the latest UN/UNICEF report, this overall reduction in child mortality rates resulted from a combination of national strategies that included the improvement of maternal and newborn care within the Unified Health System (SUS), with several actions including the creation of the Family Health Programme, the Community Health Agents Programme, and implementation of the Integrated Healthcare programs for women and for children [26]. Besides these institutional benchmarks in the health sector, many other factors can explain the advances in maternal and child health, especially the socioeconomic and demographic changes that Brazil went through during this period, with marked improvements in water and sanitation systems (especially for the poor), women’s education [25], and the implementation of important social protection policies, such as the cash transfer programme Bolsa Família [26].

As a result of all these favourable circumstances, Brazil succeeded in meeting MDGs 1 and 4 ahead of schedule with a two-thirds reduction in child mortality and a 50% reduction in the number of undernourished children between 1990 and 2015.

Among the millennium developmental goals, MDG 5 (60% reduction in maternal mortality) is one of the few goals that was not met within the established time frame and, as the results show, there are still large disparities in this process across states, a finding that is also consistent with previous reports on maternal mortality in Brazil [27–29]. It is well established that maternal morbimortality can be avoided by the provision of early access to good quality of prenatal care and good birth assistance. In Brazil, there has been a consistent increase in the coverage of antenatal care in recent decades, and as the results show, skilled birth attendance reached almost 100% in 2017. However, while the more widespread use of health services has helped to achieve important improvements in maternal morbimortality indicators, it has not fully ensured that prenatal interventions are of satisfactory quality and has led to an intensive medicalization of labour and childbirth [25]. Moreover, although maternal death cannot be attributed to one single factor, cesarean delivery rates have long been associated with multiple risks to women’s health and maternal mortality [30–34]. Cesarean is a practice that continues to increase worldwide, and it rose from 32% in 1994 to 57% in 2016 in Brazil [35]. Although the safety of cesarean delivery has improved in recent decades, it still carries potential risks to women’s health and is a modifiable risk factor of maternal mortality.

### Infectious diseases

Similarly, between 1990 and 2015, TB prevalence and mortality decreased substantially; however, the disease persists as the leading cause of death among individuals with HIV [36]. The WHO End TB strategy was developed to eliminate TB by 2035 by implementing actions to ensure that people affected by TB have proper access to diagnosis, treatment, and prevention, without facing catastrophic expenditure or worsening of their social status [37]. Nevertheless, considering the heterogeneity of TB distribution in Brazil and that the annual TB incidence is estimated to reach 31/100,000 by 2030, if the current trends remain constant, additional effort is still needed to eliminate the disease as a public health problem by 2030 [36, 38].

The 2017 GBD estimates suggest that the incidence of HIV has been increasing in all the Brazilian states. Although the risk of new infections is usually higher among vulnerable populations (e.g., men who have sex with other men, injection drug users, transgender people, or female sex workers), recent mathematical modeling studies have estimated that the overall HIV incidence has been relatively stable (i.e., increasing in men while decreasing among woman) [39] or declining in Brazil since 2010 [40, 41].

GBD data also shows that the prevalence of NTDs and WASH-related mortality (i.e., by diarrhoea, intestinal nematode infections, or protein-energy malnutrition) has also decreased in Brazil. This has been associated not only with increased sanitation and social development but could also be the result of the integration of health and social policies [42]. However, it is important to note that WASH-related mortality still affects children disproportionally [43].

### Death by external causes

It is also noteworthy that the GBD results ranked homicides rates in Brazil as one of the worst worldwide in our study period (1990–2017). In 2017, most Brazilian states were ranked as having the highest number of deaths by conflict and terrorism. However, similar to disaster mortality, deaths by conflict are too broad and hard to measure in the Brazilian context. Brazil is a highly heterogeneous country, with extreme diverse socioeconomic conditions across the regions. In addition, terrorism is inexistent in the country and therefore, it is not a good indicator for use in the Brazilian context.

The subnational estimates of GBD show that the homicide rates in Brazil remain very high, with an overall rate of 28/100,000 inhabitants, which is four times higher than the average worldwide. This rate has been increasing over the last three decades [44]. The analysis by regions and states also shows profound inequality in the distribution of these deaths. The states with the highest rates are located in the poorer regions of Brazil, reaching almost twice the overall Brazilian rate (53/100,000 inhabitants in Alagoas, 49/100,000 in Sergipe, 49/100,000 in Pernambuco), reinforcing the strong link between violent crime and poverty and incom e[45]. Homicide rates in Brazil are also related to guns availability [46], socioeconomic inequalities [44, 47, 48], and poverty [49], with specific groups such as black, men, and younger people at higher risk [44, 48]. In this context, social protection policies such as the Bolsa Familia programme have had an effect on decreasing the rates of homicide [50].

### Non-communicable diseases

The GBD estimates show that the NCD mortality rate has decreased slightly since 1990. Although NCD is still a great burden in Brazil, decreasing mortality rates have been related to the falling prevalence of smoking, widespread, and affordable delivery of drugs for the major NCD risk factors (hypertension and diabetes), the increase in the density of health workers (physicians, pharmacists, midwives or nurses), and the expansion of primary care [51, 52]. In addition, greater access to programmes for NCD prevention and control in Brazil, along with poverty reduction programmes, has been included in the agenda for NCD surveillance in Brazil [52, 53].

### Advantages and limitations

Some of the SDG targets have also been adopted by the Brazilian Institute for Applied Economic Research (IPEA); however, most of them have not been adapted for the Brazil context, either at national or subnational level [22]. Brazilian national targets include more bold targets for child stunting, child wasting, and child overweight compared to worldwide targets, and specific reductions in road injury mortality, the proportion of untreated effluent discharge, NCD and the rates of femicide and violent mortality rates of children, adolescents, young, black, indigenous, and LGBT populations. All of these targets were based on the results of the 2015 indicators [22]. Evidence of how the promotion of such goals can reduce inequalities in health comes mainly from Europe and North America.

The GBD measures have some limitations when applied to Brazilian subnational data. Although most of the indicators had similar values to national statistics, some did not match previous estimates (in particular, the incidence of HIV, hepatitis B, and malaria) [40, 41, 54, 55]. The diverse results found for HIV incidence could be due to differences in the mathematical models used to estimate the HIV incidences. GBD estimates for Brazil also showed that the incidence of hepatitis B was near 500 times higher than the one estimated by the Brazilian surveillance system in the same year (6.5/100,000) [54]. Nevertheless, the fact that GBD estimates are similar to the prevalence of the disease estimated in a systematic review of published articles from 2005–2015 [54, 56] suggests that GBD might be estimating Hepatites B prevalence rather than incidence. Finally, GBD estimates were not able to detect recent changes in malaria trends reported by the Brazilian Ministry of Health—from 2016 to 2017, the incidence of malaria increased by 59% following a long period of decline [55].

While the GBD estimates have the advantage of being produced in a timely manner, they may not be able to detect abrupt changes over time, including the economic crises in Brazil, which may have affected health-related outcomes in the country [46, 57, 58]. Finally, the fact that GBD estimates are based on several data sources not widely available to the researchers, we could not evaluate the quality of the data used to produce the Brazilian indicators.

Regarding child stunting and wasting specifically in Brazil, there are no available nationwide population-based studies that characterize the anthropometric state of children under-5. The last nutritional survey of the nutritional status of the population under-5 was made in 2007 [59–62]. Therefore, conclusions for the SDI indicators for stunting and wasting should be drawn more carefully, given their limitations in coverage and temporal proximity.

Future research is needed to evaluate the role of social and health spending in Brazil in reducing inequalities in health, contextualizing the effect of austerity policies that have been implemented in the country from 2014 onwards. The exclusive use of national surveys has been insufficient to estimate all the SDG indicators and identifies differences within Brazil. The use of subnational GBD studies is essential to estimate the subnational burden of disease in the country and to encourage the use of projections, estimates, and forecasting models [57].

## Conclusions

The majority of Brazil’s health-related SDG indicators have substantially improved in the past 28 years. However, large inequalities persist at the subnational level. The two richest regions of Brazil, the South and Southeast, had the highest SDG and SDI index in 1990 and also had the greatest rates of improvement (from 1990 to 2017). This could indicate that inequalities among states increased over this period and could negatively affect the health of the Brazilian population and maybe contribute to Brazil not achieving the SDG 2030 targets.

The GBD has been very important in providing highly comparable health data all over the world, and it should be considered as an important source of information for policy-relevant issues when the mortality and morbidity trends are relatively stable. However, GBD estimates should be always carefully interpreted if used to monitor health events as they cannot capture sudden changes as occurred in Brazil in 2015. As we have demonstrated, GBD estimates reproduce many of the GDB health indicators at the subnational level in Brazil, however, not all of them.

This study suggests that despite the great effort made to improve the major determinants of communicable and non-communicable diseases in Brazil, a continuous and stronger effort to decrease the health disparities among Brazilian states and regions remains necessary.

## Supplementary information

**Additional file 1: Figure S1.** Unscaled individual health-related indicators in Brazilian States in 2017. *The numbers represent the unscaled (or true values) of the health-related indicators for Brazil and each Brazilian State from 1990 to 2017, We omitted the colours for better visualization and added decimals for the indicators when relevant.*

**Additional file 2: Figure S2.** Performance on the health-related SDG index and individual health-related indicators in Brazilian States in 2016. *The numbers represent the rescaled indicators and combined indexes on a scale of 0 to 100, with 0 representing the worst value for each indicator or index among all 195 countries covered by GBD from 1990 to 2016, and 100 representing the best value among them within the same period. The colours represent a scale from the best (dark green) to the worse (red) indicator or index values (rows) within all Brazilian States in 2016.*

## Data Availability

Data we used in this article are publicly available online on the official website of Institute of Health Metrics and Evaluation (http://ghdx.healthdata.org/gbd-results-tool).

## Abbreviations

SDGs: Sustainable development goals
GBD: Global Burden of Disease
MDGs: Millennium Development Goals
GDP: Gross domestic product
TB: Tuberculosis
NTDs: Neglected tropical diseases
NCDs: Non-communicable diseases
UHC: Universal health coverage
IHME: Institute of Health Metrics and Evaluation
SDI index: Sociodemographic index
TFU25: Total fertility rate under the age of 25
HDI: Human Development Index
IPEA: Brazilian Institute for Applied Economic Research
