# Supplementary material for: Monitoring the progress of health-related sustainable development goals (SDGs) in Brazilian states using the Global Burden of Disease indicators
Source: Popul Health Metr. 2020 Sep 30;18(Suppl 1):7. doi: 10.1186/s12963-020-00207-2 (PMC7526114; doi:10.1186/s12963-020-00207-2)
Supplement: Supplementary file 1 — Additional file 1: Figure S1. Unscaled individual health-related indicators in Brazilian States in 2017. The numbers represent the unscaled (or true values) of the health-related indicators for Brazil and each Brazilian State from 1990 to 2017, We omitted the colours for better visualization and added decimals for the indicators when relevant. [file 12963_2020_207_MOESM1_ESM.pdf]

| Indicators | Brazil | North | | | | | | | Central-west | | | | Northeast | | | | | | | | | Southeast | | | | South | | |
|---|---|---|---|---|---|---|---|---|---|---|---|---|---|---|---|---|---|---|---|---|---|---|---|---|---|---|---|---|
| | | Acre | Amapa | Amazonas | Rondonia | Roraima | Para | Tocantins | Distrito Federal | Goias | Mato Grosso | Mato Grosso do Sul | Alagoas | Bahia | Ceara | Maranhao | Paraiba | Pernambuco | Piaui | Rio Grande do Norte | Sergipe | Espirito Santo | Minas Gerais | Sao Paulo | Rio de Janeiro | Parana | Rio Grande do Sul | Santa Catarina |
| 1.5.1: Disaster Mort | 0.0 | 0.0 | 0.0 | 0.0 | 0.0 | 0.0 | 0.0 | 0.0 | 0.0 | 0.0 | 0.0 | 0.0 | 0.0 | 0.0 | 0.0 | 0.0 | 0.0 | 0.1 | 0.0 | 0.0 | 0.0 | 0.0 | 0.0 | 0.0 | 0.0 | 0.0 | 0.0 | 0.0 |
| 2.2.1: Child Stunting | 9.7 | 13.9 | 9.5 | 11.9 | 10.4 | 7.7 | 13.6 | 11.2 | 6.3 | 9.0 | 9.0 | 7.2 | 12.8 | 11.9 | 12.7 | 17.6 | 12.2 | 11.6 | 14.4 | 9.8 | 9.8 | 7.3 | 7.6 | 6.7 | 6.8 | 7.2 | 6.5 | 4.6 |
| 2.2.2a: Child Wasting | 1.2 | 1.5 | 1.2 | 1.2 | 0.7 | 0.6 | 1.1 | 1.1 | 1.0 | 1.3 | 0.6 | 0.9 | 2.4 | 1.6 | 1.3 | 2.3 | 1.5 | 1.6 | 2.2 | 1.4 | 0.8 | 0.7 | 0.9 | 0.9 | 1.4 | 0.6 | 0.7 | 0.4 |
| 2.2.2b: Child Overweight | 31 | 28 | 30 | 29 | 27 | 28 | 24 | 24 | 49 | 33 | 33 | 30 | 29 | 25 | 28 | 20 | 26 | 28 | 25 | 27 | 26 | 31 | 29 | 36 | 36 | 35 | 37 | 38 |
| 3.1.1: Mat Mort Ratio | 67 | 60 | 87 | 94 | 88 | 50 | 72 | 68 | 54 | 62 | 78 | 90 | 57 | 65 | 51 | 66 | 53 | 56 | 70 | 47 | 65 | 59 | 61 | 68 | 103 | 77 | 80 | 47 |
| 3.1.2: Skilled Birth Attend | 99 | 97 | 100 | 97 | 100 | 99 | 98 | 98 | 100 | 100 | 100 | 100 | 98 | 98 | 98 | 97 | 99 | 98 | 96 | 99 | 100 | 100 | 100 | 100 | 100 | 100 | 100 | 100 |
| 3.2.1: Under-5 Mort | 18 | 27 | 27 | 21 | 19 | 22 | 21 | 20 | 14 | 18 | 20 | 18 | 21 | 23 | 19 | 21 | 15 | 20 | 20 | 16 | 21 | 17 | 16 | 16 | 18 | 17 | 15 | 16 |
| 3.2.2: Neonatal Mort | 8.5 | 13.6 | 15.4 | 10.1 | 9.6 | 9.4 | 9.0 | 10.2 | 8.8 | 10.3 | 9.7 | 8.7 | 7.8 | 8.6 | 7.5 | 7.8 | 5.6 | 8.1 | 10.6 | 5.1 | 10.3 | 8.9 | 7.8 | 8.4 | 9.1 | 8.1 | 8.1 | 8.2 |
| 3.3.1: HIV Incid | 0.4 | 0.2 | 0.3 | 0.5 | 0.2 | 0.2 | 0.5 | 0.2 | 0.2 | 0.2 | 0.3 | 0.3 | 0.3 | 0.3 | 0.3 | 0.4 | 0.2 | 0.3 | 0.3 | 0.2 | 0.2 | 0.2 | 0.2 | 0.2 | 0.4 | 0.2 | 0.4 | 0.3 |
| 3.3.2: TB Incid | 36 | 61 | 51 | 80 | 28 | 45 | 47 | 23 | 23 | 19 | 42 | 37 | 32 | 37 | 39 | 40 | 29 | 47 | 35 | 33 | 30 | 26 | 26 | 32 | 55 | 22 | 41 | 23 |
| 3.3.3: Malaria Incid | 1.0 | 33.0 | 23.4 | 20.3 | 13.3 | 27.2 | 3.7 | 0.0 | 0.0 | 0.0 | 0.5 | 0.0 | 0.0 | 0.0 | 0.0 | 0.3 | 0.0 | 0.0 | 0.0 | 0.0 | 0.0 | 0.0 | 0.0 | 0.0 | 0.0 | 0.0 | 0.0 | 0.0 |
| 3.3.4: Hep B Incid | 1259 | 2002 | 1151 | 1446 | 1410 | 1549 | 1502 | 1102 | 1122 | 1139 | 1154 | 1173 | 1513 | 1229 | 1134 | 1322 | 1414 | 1493 | 1238 | 1213 | 1377 | 997 | 1167 | 1261 | 1215 | 1250 | 1232 | 1158 |
| 3.3.5: NTD Prev | 10.1 | 7.4 | 6.2 | 6.3 | 10.9 | 6.2 | 11.2 | 6.8 | 24.7 | 16.4 | 5.9 | 5.8 | 9.8 | 8.8 | 6.3 | 9.4 | 7.3 | 12.7 | 8.1 | 5.9 | 7.7 | 6.3 | 18.1 | 11.3 | 6.7 | 5.8 | 4.8 | 5.1 |
| 3.4.1: NCD Mort | 330 | 293 | 307 | 289 | 312 | 290 | 307 | 286 | 247 | 306 | 297 | 339 | 365 | 329 | 266 | 311 | 329 | 384 | 283 | 301 | 313 | 293 | 306 | 335 | 398 | 336 | 352 | 305 |
| 3.4.2: Suicide Mort | 6.0 | 6.5 | 6.4 | 6.2 | 6.4 | 8.3 | 3.9 | 7.1 | 4.0 | 6.8 | 5.6 | 8.0 | 3.7 | 5.2 | 6.7 | 3.9 | 5.0 | 5.3 | 7.8 | 7.2 | 6.8 | 4.0 | 7.1 | 5.3 | 4.2 | 6.7 | 10.3 | 8.3 |
| 3.5.2: Alcohol Use | 17 | 14 | 15 | 15 | 16 | 15 | 14 | 15 | 20 | 17 | 17 | 17 | 15 | 16 | 16 | 14 | 16 | 16 | 15 | 17 | 16 | 18 | 17 | 19 | 19 | 18 | 19 | 18 |
| 3.6.1: Road Inj Mort | 20 | 17 | 18 | 14 | 30 | 29 | 20 | 34 | 17 | 26 | 29 | 24 | 22 | 19 | 23 | 21 | 23 | 22 | 30 | 20 | 26 | 22 | 20 | 15 | 19 | 25 | 17 | 24 |
| 3.7.1: FP Need Met, Mod | 88 | 87 | 89 | 87 | 89 | 88 | 85 | 86 | 90 | 94 | 91 | 92 | 83 | 83 | 82 | 82 | 84 | 84 | 85 | 88 | 85 | 92 | 86 | 90 | 91 | 86 | 91 | 90 |
| 3.7.2: Adol Birth Rate | 27 | 39 | 33 | 41 | 29 | 42 | 37 | 36 | 20 | 27 | 29 | 32 | 34 | 28 | 24 | 47 | 27 | 31 | 28 | 28 | 26 | 24 | 20 | 22 | 25 | 25 | 22 | 19 |
| 3.9.1: Air Poll Mort | 24 | 25 | 21 | 20 | 26 | 23 | 25 | 25 | 33 | 35 | 26 | 24 | 24 | 23 | 18 | 24 | 21 | 24 | 21 | 17 | 19 | 19 | 24 | 25 | 31 | 22 | 24 | 21 |
| 3.9.2: WaSH Mort | 3.6 | 6.0 | 4.0 | 6.2 | 5.2 | 6.0 | 6.1 | 3.4 | 1.7 | 2.5 | 3.4 | 4.1 | 7.4 | 5.0 | 4.8 | 5.6 | 4.3 | 5.8 | 5.0 | 4.2 | 4.7 | 2.1 | 2.6 | 2.3 | 2.8 | 2.7 | 1.9 | 2.2 |
| 3.9.3: Poisoning Mort | 0.2 | 0.2 | 0.2 | 0.2 | 0.3 | 0.3 | 0.2 | 0.3 | 0.2 | 0.2 | 0.3 | 0.2 | 0.1 | 0.2 | 0.1 | 0.2 | 0.2 | 0.1 | 0.2 | 0.2 | 0.2 | 0.1 | 0.2 | 0.1 | 0.3 | 0.2 | 0.2 | 0.1 |
| 3.a.1: Smoking Prev | 9.5 | 9.3 | 7.5 | 6.7 | 8.7 | 7.3 | 7.2 | 7.2 | 9.5 | 8.8 | 9.1 | 10.7 | 6.8 | 5.8 | 6.8 | 5.9 | 7.1 | 8.6 | 6.8 | 7.1 | 6.2 | 7.6 | 9.8 | 12.2 | 9.7 | 12.4 | 12.8 | 10.2 |
| 3.b.1: Vaccine Cov | 92 | 85 | 87 | 91 | 97 | 93 | 82 | 92 | 98 | 93 | 95 | 95 | 89 | 88 | 95 | 86 | 88 | 93 | 86 | 88 | 91 | 94 | 94 | 94 | 94 | 95 | 92 | 97 |
| 3.c.1: Health Worker Dens | 8.1 | 4.0 | 6.7 | 5.3 | 3.2 | 6.4 | 5.2 | 5.9 | 11.7 | 7.2 | 6.9 | 7.8 | 5.2 | 5.6 | 5.3 | 5.4 | 5.2 | 7.3 | 5.1 | 8.9 | 6.0 | 8.7 | 8.9 | 10.2 | 13.1 | 6.6 | 10.4 | 7.5 |
| 5.2.1: Int Partner Viol | 7.2 | 7.9 | 7.6 | 7.6 | 7.6 | 7.7 | 8.2 | 7.5 | 5.7 | 7.3 | 7.2 | 7.3 | 8.7 | 8.0 | 7.9 | 9.0 | 8.0 | 7.9 | 8.2 | 7.7 | 7.7 | 7.0 | 6.9 | 6.3 | 6.8 | 6.9 | 6.6 | 6.3 |
| 5.2.2: Non-Int Partner Sex Viol | 0.4 | 0.4 | 0.5 | 0.4 | 0.4 | 0.5 | 0.4 | 0.4 | 0.5 | 0.5 | 0.5 | 0.5 | 0.4 | 0.4 | 0.4 | 0.4 | 0.4 | 0.4 | 0.4 | 0.4 | 0.4 | 0.5 | 0.5 | 0.3 | 0.5 | 0.5 | 0.5 | 0.5 |
| 6.1.1: Water | 9.8 | 24.4 | 9.5 | 17.0 | 23.2 | 9.2 | 25.5 | 12.3 | 3.9 | 8.5 | 10.5 | 7.7 | 15.7 | 12.4 | 14.6 | 23.3 | 12.7 | 12.2 | 14.9 | 9.8 | 9.8 | 7.1 | 7.6 | 3.8 | 6.7 | 6.1 | 7.0 | 7.5 |
| 6.2.1a: Sanitation | 23.3 | 43.2 | 51.2 | 39.7 | 63.1 | 47.7 | 54.3 | 54.0 | 5.4 | 29.4 | 45.1 | 42.6 | 44.7 | 30.6 | 36.3 | 56.7 | 32.2 | 30.2 | 56.9 | 36.7 | 27.4 | 14.1 | 10.9 | 4.7 | 7.9 | 17.2 | 19.1 | 18.7 |
| 6.2.1b: Hygiene | 19 | 29 | 19 | 24 | 27 | 19 | 30 | 21 | 14 | 17 | 19 | 17 | 23 | 21 | 22 | 30 | 21 | 21 | 24 | 19 | 19 | 16 | 17 | 14 | 16 | 16 | 16 | 16 |
| 7.1.2: HH Air Poll | 5.6 | 6.5 | 2.6 | 4.8 | 8.2 | 3.1 | 9.2 | 8.7 | 0.2 | 2.8 | 4.7 | 4.4 | 10.6 | 10.2 | 9.4 | 13.4 | 11.0 | 8.2 | 12.4 | 7.6 | 7.8 | 5.8 | 6.0 | 0.9 | 1.2 | 5.6 | 7.1 | 7.0 |
| 8.8.1: Occ Burden | 704 | 803 | 652 | 737 | 748 | 701 | 752 | 687 | 502 | 690 | 678 | 692 | 732 | 778 | 705 | 794 | 724 | 763 | 742 | 679 | 687 | 640 | 684 | 701 | 576 | 695 | 733 | 721 |
| 11.6.2: Mean PM2.5 | 12.7 | 13.4 | 13.4 | 12.4 | 13.2 | 11.5 | 13.0 | 19.0 | 37.6 | 30.6 | 17.7 | 11.5 | 7.4 | 10.6 | 9.0 | 11.8 | 7.4 | 7.6 | 10.8 | 7.8 | 7.2 | 10.4 | 14.2 | 13.5 | 13.6 | 9.2 | 10.2 | 9.7 |
| 16.1.1: Homicide | 28 | 30 | 36 | 35 | 35 | 28 | 37 | 27 | 23 | 38 | 32 | 24 | 53 | 34 | 35 | 23 | 35 | 46 | 19 | 45 | 49 | 35 | 23 | 14 | 35 | 27 | 26 | 12 |
| 16.1.2: Conflict Mort | 0.0 | 0.0 | 0.0 | 0.0 | 0.0 | 0.0 | 0.0 | 0.0 | 0.0 | 0.0 | 0.0 | 0.0 | 0.0 | 0.0 | 0.0 | 0.0 | 0.0 | 0.0 | 0.0 | 0.0 | 0.0 | 0.0 | 0.0 | 0.0 | 0.0 | 0.0 | 0.0 | 0.0 |
| 16.1.3a: Physical Violence | 4.4 | 4.4 | 4.4 | 4.4 | 4.4 | 4.4 | 4.4 | 4.4 | 4.4 | 4.4 | 4.4 | 4.4 | 4.4 | 4.4 | 4.4 | 4.4 | 4.4 | 4.4 | 4.4 | 4.4 | 4.4 | 4.4 | 4.4 | 4.4 | 4.4 | 4.4 | 4.4 | 4.4 |
| 16.1.3c: Sexual Violence | 1.8 | 1.8 | 1.8 | 1.8 | 1.7 | 1.8 | 1.8 | 1.7 | 1.9 | 1.8 | 1.8 | 1.7 | 1.7 | 1.7 | 1.8 | 1.7 | 1.7 | 1.8 | 1.7 | 1.8 | 1.8 | 1.8 | 1.7 | 1.8 | 1.8 | 1.8 | 1.8 | 1.8 |
| 16.2.3: Child Sex Abuse | 6.2 | 5.9 | 5.9 | 5.9 | 5.9 | 5.9 | 6.8 | 5.9 | 6.0 | 3.7 | 5.9 | 5.9 | 6.0 | 3.5 | 7.2 | 4.4 | 6.0 | 6.3 | 6.0 | 6.0 | 6.0 | 5.9 | 5.9 | 8.3 | 4.8 | 8.6 | 2.7 | 7.7 |
| 17.19.2c: Cert Death Reg | 83 | 82 | 82 | 80 | 82 | 84 | 80 | 85 | 91 | 86 | 84 | 89 | 82 | 73 | 80 | 80 | 80 | 83 | 82 | 84 | 82 | 90 | 80 | 85 | 81 | 86 | 86 | 86 |
