# Supplementary material for: Monitoring the progress of health-related sustainable development goals (SDGs) in Brazilian states using the Global Burden of Disease indicators
Source: Popul Health Metr. 2020 Sep 30;18(Suppl 1):7. doi: 10.1186/s12963-020-00207-2 (PMC7526114; doi:10.1186/s12963-020-00207-2)
Supplement: Supplementary file 2 — Additional file 2: Figure S2. Performance on the health-related SDG index and individual health-related indicators in Brazilian States in 2016. The numbers represent the rescaled indicators and combined indexes on a scale of 0 to 100, with 0 representing the worst value for each indicator or index among all 195 countries covered by GBD from 1990 to 2016, and 100 representing the best value among them within the same period. The colours represent a scale from the best (dark green) to the worse (red) indicator or index values (rows) within all Brazilian States in 2016. [file 12963_2020_207_MOESM2_ESM.pdf]

Indicators	Brazil	North							Central-west				Northeast									Southeast				South		
		Acre	Amapa	Amazonas	Rondonia	Roraima	Para	Tocantins	Distrito Federal	Goias	Mato Grosso	Mato Grosso do Sul	Alagoas	Bahia	Ceara	Maranhao	Paraiba	Pernambuco	Piaui	Rio Grande do Norte	Sergipe	Espirito Santo	Minas Gerais	Sao Paulo	Rio de Janeiro	Parana	Rio Grande do Sul	Santa Catarina
**SDG Index**	**66**	**62**	**61**	**62**	**61**	**64**	**62**	**63**	**66**	**63**	**63**	**66**	**64**	**65**	**66**	**63**	**67**	**66**	**64**	**68**	**66**	**69**	**68**	**68**	**66**	**67**	**67**	**67**
1.5.1: Disaster Mort	**66**	80	37	51	80	80	73	80	80	72	66	80	80	65	74	67	80	80	80	67	80	80	65	59	61	80	80	58
2.2.1: Child Stunting	**86**	78	86	81	84	89	78	83	92	87	87	90	80	81	80	71	81	82	77	85	85	90	90	91	91	90	92	95
2.2.2a: Child Wasting	**97**	95	97	97	100	100	98	97	98	96	100	99	90	95	96	91	95	95	91	96	99	100	99	99	96	100	100	100
2.2.2b: Child Overweight	**29**	37	32	33	41	38	47	47	1	24	23	33	33	44	36	60	42	36	46	39	42	28	33	17	16	19	15	12
3.1.1: Mat Mort Ratio	**43**	45	37	35	37	48	41	41	47	44	38	37	47	44	49	43	46	48	42	50	44	45	45	42	35	41	41	50
3.1.2: Skilled Birth Attend	**99**	96	100	96	100	99	97	97	100	100	100	100	98	97	97	96	99	97	95	98	100	100	100	100	100	100	100	100
3.2.1: Under-5 Mort	**52**	44	43	48	51	47	49	51	58	53	50	52	50	47	52	50	58	51	51	57	49	54	56	56	53	54	57	56
3.2.2: Neonatal Mort	**47**	34	29	41	42	43	45	42	46	41	42	46	49	47	50	49	59	48	41	61	41	45	49	46	44	48	47	48
3.3.1: HIV Incid	**38**	49	43	35	46	44	35	45	48	45	40	42	40	43	41	37	43	40	41	48	44	44	46	46	37	43	37	40
3.3.2: TB Incid	**62**	52	56	47	67	58	57	71	72	75	59	62	64	62	60	61	66	57	63	64	66	67	69	64	54	71	59	70
3.3.3: Malaria Incid	**32**	11	13	14	16	12	24	52	60	58	36	61	70	71	67	39	70	72	56	64	67	57	65	64	64	62	71	66
3.3.4: Hep B Incid	**53**	37	56	48	49	46	47	58	57	56	56	55	47	54	57	52	49	47	54	55	50	61	56	53	54	53	54	56
3.3.5: NTD Prev	**90**	92	93	93	88	93	88	93	75	83	94	94	90	91	93	90	92	87	92	94	92	93	81	88	93	94	95	95
3.4.1: NCD Mort	**64**	70	68	70	66	70	68	72	80	66	69	62	57	64	74	66	63	56	72	69	66	70	68	63	54	63	61	68
3.4.2: Suicide Mort	**69**	66	67	66	66	54	88	62	85	63	72	57	89	76	62	86	76	75	57	63	63	85	62	73	86	64	47	55
3.5.2: Alcohol Use	**47**	55	53	53	51	54	55	52	37	48	48	49	55	52	52	57	52	52	54	49	52	45	46	42	43	45	43	44
3.6.1: Road Inj Mort	**36**	43	41	47	21	23	36	17	42	26	22	29	32	39	29	33	30	33	20	37	26	32	36	47	40	28	42	30
3.7.1: FP Need Met, Mod	**97**	96	98	96	98	97	93	95	99	100	99	100	91	90	89	89	92	92	93	96	93	100	94	99	99	94	99	99
3.7.2: Adol Birth Rate	**31**	20	26	19	30	18	22	23	40	31	29	26	24	29	34	14	30	28	30	30	32	34	40	37	34	35	38	41
3.9.1: Air Poll Mort	**65**	65	70	73	64	69	64	65	55	53	63	67	65	68	76	66	69	67	71	78	72	72	66	64	59	69	67	69
3.9.2: WaSH Mort	**52**	45	51	44	47	45	45	53	62	57	53	51	42	48	48	46	50	46	48	50	49	59	57	59	56	56	61	59
3.9.3: Poisoning Mort	**87**	81	85	82	73	70	87	74	88	82	74	78	100	78	91	81	89	93	81	91	78	94	89	96	77	88	90	94
3.a.1: Smoking Prev	**85**	86	92	95	88	93	93	93	85	88	87	81	94	97	94	97	93	88	94	93	96	92	84	76	85	76	74	83
3.b.1: Vaccine Cov	**95**	86	88	93	100	96	82	95	100	95	97	98	92	90	98	87	90	96	87	91	93	97	97	97	97	98	94	100
3.c.1: Health Worker Dens	**90**	60	77	72	66	78	74	76	96	87	85	89	67	78	77	70	78	82	69	88	81	91	92	94	97	87	93	88
5.2.1: Int Partner Viol	**88**	85	86	86	86	86	84	86	93	87	87	87	82	84	85	81	84	85	84	86	86	88	88	91	89	89	89	90
5.2.2: Non-Int Partner Sex Viol	**88**	88	87	88	88	88	89	88	85	88	87	88	89	89	88	90	89	88	89	88	88	87	87	91	86	87	87	87
6.1.1: Water	**89**	71	89	80	73	89	70	86	96	90	88	91	82	86	83	72	85	86	83	89	89	92	91	96	92	93	92	91
6.2.1a: Sanitation	**74**	52	43	56	30	47	40	40	95	67	50	53	50	66	59	37	64	66	37	59	70	85	88	95	92	81	79	79
6.2.1b: Hygiene	**83**	71	82	77	74	82	70	80	88	84	83	84	77	80	79	70	80	80	77	82	82	85	85	87	86	86	85	86
7.1.2: HH Air Poll	**92**	91	96	93	89	96	87	88	100	96	94	94	85	86	87	81	85	89	83	90	89	92	92	99	98	92	90	90
8.8.1: Occ Burden	**55**	46	59	52	51	54	50	56	76	56	57	56	52	49	55	47	53	50	52	57	56	61	57	55	68	56	52	53
11.6.2: Mean PM2.5	**73**	75	74	80	76	81	75	65	33	43	67	79	92	82	87	81	91	90	83	91	93	81	70	71	71	87	82	84
16.1.1: Homicide	**7**	5	1	1	2	6	0	7	10	0	3	11	0	2	0	10	1	0	16	0	0	1	11	23	3	8	9	26
16.1.2: Conflict Mort	**91**	100	100	100	100	100	100	100	100	100	100	100	100	100	100	66	100	100	100	100	100	100	100	100	100	100	100	100
16.1.3a: Physical Violence	**85**	85	85	85	85	85	85	85	85	85	85	85	85	85	85	85	85	85	85	85	85	85	85	85	85	85	85	85
16.1.3c: Sexual Violence	**80**	80	78	78	81	79	80	81	76	80	80	80	81	81	80	81	81	80	81	80	80	80	80	78	79	80	80	80
16.2.3: Child Sex Abuse	**77**	79	79	79	79	79	74	79	78	89	79	79	78	91	72	86	78	76	78	78	78	78	79	67	84	65	94	69
17.19.2c: Cert Death Reg	**89**	88	88	86	89	91	87	92	98	93	91	96	88	79	87	86	87	90	89	90	89	97	86	92	87	92	93	93
